# 快速现场评估的细胞学涂片对非小细胞肺癌分子检测的补充作用

**DOI:** 10.3779/j.issn.1009-3419.2023.101.35

**Published:** 2023-12-20

**Authors:** Shiqi TANG, Chunli TANG, Zeyun LIN, Juhong JIANG

**Affiliations:** 510120 广州，广州医科大学附属第一医院，广州呼吸健康研究院，呼吸疾病国家重点实验室/国家呼吸系统疾病临床医学研究中心; The State Key Laboratory of Respiratory Disease, Guangzhou Institute of Respiratory Health, National Clinical Research Center for Respiratory Disease, The First Affiliated Hospital, Guangzhou Medical University, Guangzhou 510120, China

**Keywords:** 肺肿瘤, 活检, 快速现场细胞学评估, 分子检测, Lung neoplasms, Biopsy, Rapid on-site evaluation, Molecular testing

## Abstract

**背景与目的** 胸部小活检标本取材包括经支气管镜肺活检术（transbronchial forceps lung biopsy, TBLB）以及超声内镜引导下的经支气管针吸活检（endobronchial ultrasound transbronchial needle aspiration, EBUS-TBNA）可采用术中快速现场评估（rapid on-site evaluation, ROSE）细胞学涂片为操作者迅速给出初步诊断。本研究旨在分析ROSE细胞学涂片对非小细胞肺癌（non-small cell lung cancer, NSCLC）分子检测的补充作用。**方法** 回顾性分析2020年8月至2022年12月我院收治的308例TBLB及EBUS-TBNA活检并行术中ROSE、病理诊断为NSCLC的病例，评估分析了配对的石蜡切片及ROSE细胞学涂片的肿瘤细胞量和比例，提取细胞学涂片的DNA并定量，部分合格的细胞学涂片标本用于实时定量聚合酶链式反应（polymerase chain reaction, PCR）和第二代测序（next-generation sequencing, NGS）检测。**结果** 大部分ROSE细胞学涂片均具有丰富的肿瘤细胞，78例（25.3%）石蜡切片分子检测不合格的病例中有44例（14.3%）具有分子检测合格的细胞涂片。石蜡切片检测到的细胞突变均可在合格的细胞学涂片标本中检测到。**结论** 胸部小活检标本的ROSE细胞学涂片标本对NSCLC的分子检测有很好的补充作用，细胞学涂片与传统的石蜡切片联合应用能促进晚期NSCLC患者靶向治疗驱动基因的检测。建议实验室在评估石蜡切片分子检测不合格时，可进一步评估患者的ROSE细胞学涂片，合格的细胞学涂片可作为NSCLC分子检测的补充标本。

## Abstract (en)

**Background and objective** The thoracic small biopsy sampling procedure including transbronchial forceps lung biopsy (TBLB) and endobronchial ultrasound transbronchial needle aspiration (EBUS-TBNA) can be accompanied by rapid on-site evaluation (ROSE) of sample material to provide immediate feedback for the proceduralist. The present study aims to investigate the supplemental effect of ROSE smear samples for lung cancer molecular test. **Methods** In a retrospective study, 308 patients admitted to our hospital from August 2020 to December 2022 undergoing diagnostic TBLB and EBUS-TBNA with ROSE and subsequently diagnosed as non-small cell lung cancer (NSCLC) were analyzed. The matched formalin-fixed paraffin-embedding (FFPE) tissue section and ROSE smears for tumor cellularity were compared. DNA yields of smears were determined. Real-time polymerase chain reaction (PCR) and next-generation sequencing (NGS) were performed on adequate smear samples. **Results** ROSE smear samples were enriched in tumor cells. Among 308 biopsy samples, 78 cases (25.3%) exhibited inadequate FFPE tissue sections, whereas 44 cases (14.3%) yielded adequate smear samples. Somatic mutations detected in the FFPE tissue section samples were also detected in the matching adequate smear sample. **Conclusion** ROSE smear samples of the thoracic small biopsies are beneficial supplemental materials for ancillary testing of lung cancer. Combined use of cytology smear samples with traditional FFPE section samples can enhance the detection rate of informative mutations in patients with advanced NSCLC. We recommend that the laboratory could further evaluate the ROSE cell smears of the patient when FFPE tissue sections are inadequate, and that adequate cell smears can be used as a supplemental source for the molecular testing of NSCLC.

肺癌是世界上最常见的恶性肿瘤，也是癌症相关死亡的主要原因^[1]^。非小细胞肺癌（non-small cell lung cancer, NSCLC）是最常见的肺癌组织学类型，约占肺癌病例的80%^[2]^。约70%的肺癌患者因诊断时病情已处于晚期或伴有转移而不能手术，只能通过小活检组织或细胞学标本进行诊断^[3]^。随着NSCLC驱动基因研究的进展，对于新诊断的晚期NSCLC，需要检测的治疗相关标志物将会进一步增加^[1⇓-3]^，临床对小活检标本和细胞学标本辅助分子检测的需求将持续增长。目前普遍认为福尔马林固定的石蜡包埋（formalin-fixed paraffin-embedding, FFPE）组织切片是较为可靠的肿瘤分子检测材料。然而，部分石蜡包埋组织较少，有时进行组织学诊断或补充性的免疫组化检测之后没有足够的肿瘤细胞进行分子检测，重复活检又将增加患者的负担。现有的研究^[4]^报道肺癌小活检标本进行第二代测序（next-generation sequencing, NGS）的标本合格率只有60%-80%。虽然液态活检对分子检测有一些补充作用，但是存在30%左右的假阴性^[5,6]^。寻找可靠的组织来源以完成多种检测方法仍然是临床病理的一大挑战。

快速现场评估（rapid on-site evaluation, ROSE）是指在介入活检操作过程中，对现场获得的小活检或细胞学标本立即进行细胞学制片、染色、评估，以初步判断取材是否成功及指导下一步操作和标本处理^[7,8]^。术中ROSE可提高穿刺成功率，减少穿刺次数，降低并发症。有研究与指南^[9⇓⇓-12]^推荐，NSCLC分子检测标本可以采用细胞蜡块也可以是其他细胞学标本（直接涂片或液基细胞学涂片）。本研究回顾性分析了我院经支气管镜肺活检术（transbronchial forceps lung biopsy, TBLB）或者超声内镜引导下的经支气管针吸活检（endobronchial ultrasound transbronchial needle aspiration, EBUS-TBNA）并进行了术中ROSE的NSCLC病例。采用显微镜观察评估所有病例石蜡切片及其配对的ROSE细胞学涂片肿瘤细胞量及比例，以探讨ROSE细胞学涂片标本对NSCLC分子检测的补充作用。

## 1 资料与方法

### 1.1 临床病例收集

纳入2020年8月至2022年12月于广州医科大学附属第一医院呼吸介入科行TBLB及EBUS-TBNA活检且术中行ROSE病例。纳入病例的小活检标本均由本院呼吸病理中心完成常规病理学检查并最终诊断为NSCLC。经我院的临床信息系统及病理信息系统查询并收集每例患者的年龄、性别、标本来源及其取材方法以及免疫组织化学结果等信息，调取每例患者的石蜡切片和ROSE细胞涂片进行质量评估。本研究经广州医科大学第一附属医院机构审查委员会批准（审批编号：2021-70；批准日期：2021年8月16日）。

### 1.2 ROSE细胞涂片制作方法

TBLB获取标本后，用镊子夹住小活检组织在载玻片上的1-2 cm的椭圆形区域顺时针方向滚涂（组织印片）。EBUS-TBNA获取标本后，用针芯推出组织，并用抽满空气的注射器再次吹出套管针内残留的液体，滴一滴于载玻片上，用另一块玻片垂直方向推动液体（涂片）。每例患者至少制作两张涂片，涂片后立即采用迪夫染液对空气晾干的细胞学涂片进行快速染色，染液系按照世界卫生组织（World Health Organization, WHO）推荐的快速染色方法配制^[13]^。如果前面两次活检操作为阳性，后面两次操作获取的组织全部留作石蜡切片。

### 1.3 石蜡切片和ROSE细胞涂片的肿瘤细胞量评估

纳入的病例均有配对的石蜡组织切片和ROSE细胞学涂片。所有病例石蜡组织进行诊断性免疫组化连续切片，对最后一张石蜡切片的肿瘤细胞数量和比例进行评估。在光学显微镜下观察整张切片的每一块小组织，或将切片分为若干个区域，依次计数每小块组织或区域的肿瘤细胞及正常细胞数量，计算肿瘤细胞总的数量及比例。ROSE细胞学涂片的评估采用光学显微镜从左至右依次观察涂片所有区域，计算肿瘤细胞总的数量及比例。

### 1.4 ROSE细胞涂片DNA提取

由本研究的实验人员采用QIAGEN DNA提取试剂盒（DNeasy Blood and Tissue Kit）提取ROSE细胞涂片标本DNA，具体步骤如下：两张ROSE涂片分别滴90 μL Buffer ATL，覆盖玻片上有细胞的区域，用一次性手术刀片刮下细胞，聚拢液体成分，用移液器吸取液体置于1.5 mL离心管中，加入20 μL蛋白激酶K，振荡混匀，56^o^C孵育至组织完全裂解后按照试剂盒说明书的操作流程进行，最后采用100 μL Buffer AE洗脱获得DNA，采用超微量分光光度计测量DNA浓度后储存于-20 ^o^C冰箱中。

### 1.5 实时荧光定量聚合酶链式反应（polymerase chain reaction, PCR）技术及高通量NGS检测分子突变

本院临床诊疗过程中检测NSCLC患者靶基因突变最常用的两种平台有厦门艾德生物医药科技股份有限公司的肺癌9个基因突变实时定量PCR联合检测试剂盒和北京吉因加检验所的包含1021个基因的高通量NGS套餐^[14,15]^。两种检测方法均包含美国国立综合癌症网络（National Comprehensive Cancer Network, NCCN）指南推荐检测的9个基因的突变：表皮生长因子受体（epidermal growth factor receptor, EGFR）、BRAF、ERBB2、KRAS基因突变，间变性淋巴瘤激酶（anaplastic lymphoma kinase, ALK）、ROS1、RET、NTRK1/2/3基因融合、MET基因扩增和MET基因14号外显子跳跃突变。实时定量PCR要求的最低DNA量是10 ng，NGS要求的最低DNA量是50 ng。

### 1.6 统计学方法

数据采用SPSS 25.0软件进行分析和处理。采用斯皮尔曼相关性分析ROSE涂片肿瘤细胞量与DNA提取总量的关系。P<0.05为差异有统计学意义。

## 2 结果

### 2.1 临床特征

本研究纳入308例患者，中位年龄64岁（30-85岁），其中男性210例，女性98例；TBLB取材标本210例，EBUS-TBNA取材标本98例。病例均经石蜡切片及免疫组织化学诊断为NSCLC：其中腺癌196例，鳞状细胞癌88例，非特殊类型癌24例。

### 2.2 石蜡组织和ROSE细胞涂片的肿瘤细胞数量及比例

根据每张石蜡切片的肿瘤细胞数量和比例，将石蜡组织标本分为两组：不合格组：每张切片<200个肿瘤细胞或肿瘤细胞比例<10%；合格组：每张切片≥200个肿瘤细胞且肿瘤细胞比例≥10%。根据每例患者所有ROSE细胞涂片的肿瘤细胞总量和比例对ROSE细胞涂片标本进行分组。低量：患者所有ROSE涂片细胞总量<1000个肿瘤细胞或肿瘤细胞比例<10%，不满足分子检测条件；中量：患者所有ROSE涂片细胞总量为1000-3000个肿瘤细胞且肿瘤细胞比例≥10%，满足分子检测条件；高量：每例患者所有ROSE涂片细胞总量>3000个肿瘤细胞且肿瘤细胞比例≥10%，非常适合于分子检测。在308例ROSE涂片标本中，低量组98例，中量组146例，高量组64例；分析发现TBLB组和EBUS-TBNA组ROSE细胞涂片分子检测合格率分别为76.2%和51.0%（表1）。

**表1:** TBLB和EBUS-TBNA标本的配对的FFPE切片和ROSE肿瘤细胞量的分组详细情况

Detecting methods	FFPE			ROSE smears		
				Low	Moderate	High
TBLB	Inadequate	61 (29.0%)		20 (32.8%)	30 (49.2%)	11 (18.0%)
	Adequate	149 (71.0%)		30 (20.1%)	81 (54.4%)	38 (25.5%)
	Total	210		50 (23.8%)	111 (52.9%)	49 (23.3%)
EBUS-TBNA	Inadequate	17 (17.3%)		14 (82.4%)	2 (11.8%)	1 (5.8%)
	Adequate	81 (82.7%)		34 (42.0%)	33 (40.7%)	14 (17.3%)
	Total	98		48 (49.0%)	35 (35.7%)	15 (15.3%)

FFPE cellularity threshold: Inadequate, with <200 tumor cells/section and/or a tumor cell fraction <10%, inadequate for molecular testing; Adequate, with ≥200 tumor cells/section and a tumor cell fraction ≥10%, adequate for molecular testing. ROSE cellularity threshold: Low, with <1000 tumor cells/smear and/or a tumor cell fraction <10%, inadequate for molecular testing; Moderate, with 1000-3000 tumor cells/smear and a tumor cell fraction ≥10%, adequate for molecular testing; High, with >3000 tumor cells/smear and a tumor cell fraction ≥10%, advantageous for molecular testing. TBLB: transbronchial lung biopsy; EBUS-TBNA: endobronchial ultrasound-guided transbronchial needle aspiration; FFPE: formalin-fixed paraffin-embedded; ROSE: rapid on-site evaluation.

### 2.3 ROSE细胞涂片样本对石蜡切片分子检测合格率的补充作用

在210例TBLB标本中，两种标本结合使用的情况下，合格率由71.0%提高至90.5%。在98例EBUS-TBNA标本中，两种标本结合使用的情况下，合格率由82.7%提高至85.7%（表2）。图1展示了石蜡包埋切片与ROSE的细胞学特征。

**表2:** ROSE细胞涂片样本对FFPE切片分子检测合格率的补充作用

Detecting methods	FFPE adequacy rate	Adequacy increase by adding adequate ROSE	Combined adequacy rate
TBLB	71.0% (149/210)	19.5% (41/210)	90.5% (190/210)
EBUS-TBNA	82.7% (81/98)	3.1% (3/98)	85.7% (84/98)
Total	74.7% (230/308)	14.3% (44/308)	89.0% (274/308)

**图1:**
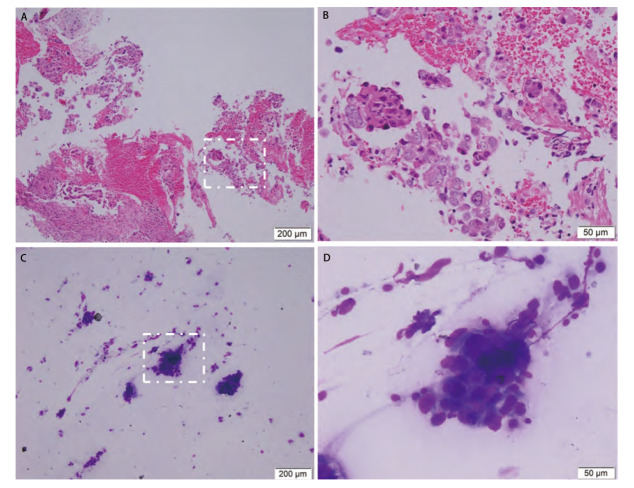
FFPE切片的组织学特征与ROSE的细胞学特征。A、B：仅少量供诊断的肿瘤细胞存在于HE染色的石蜡包埋切片；C、D：较多肿瘤细胞存在于迪夫染色的ROSE组织印片。

### 2.4 ROSE细胞涂片标本的DNA提取量

TBLB组210例ROSE细胞涂片提取的DNA量中位数为17.4 ng（0-423.7 ng），DNA量>10 ng（实时定量PCR检测要求的最低DNA量）的病例占74.3%（156/210），>50 ng（NGS检测要求的最低DNA量）的病例占16.7%（35/210）。EBUS-TBNA组98例ROSE细胞涂片提取的DNA量中位数为12.65 ng（0-158.4 ng），DNA量>10 ng的病例占61.2%（60/98），>50 ng的病例占9.2%（9/98）。采用斯皮尔曼相关性分析ROSE涂片肿瘤细胞量与DNA提取总量的关系，结果表明，ROSE涂片肿瘤细胞量与DNA提取总量具有显著相关性（相关系数rho值=0.792，P<0.001），具有统计学意义，ROSE细胞涂片肿瘤细胞量高，提取的DNA总量高（表3）。

**表3:** ROSE细胞涂片肿瘤细胞量与DNA总量的相关性

Detecting methods	Cellularity of ROSE smears	DNA yield of ROSE smears			
		Total	<10 ng	10-50 ng	>50 ng
TBLB	<1000	50	37	12	1
	1000-3000	111	17	86	8
	>3000	49	0	23	26
EBUS-TBNA	<1000	48	34	14	0
	1000-3000	35	4	28	3
	>3000	15	0	9	6

### 2.5 分子检测结果

在标本收集过程中，有92例患者的石蜡组织标本遵照医嘱进行了肺癌9个基因突变实时定量PCR联合检测，有38例患者的石蜡组织标本遵照医嘱进行了NGS检测，我们从这些病例配对ROSE细胞涂片标本中为两种检测方法各挑选了5例肿瘤细胞量合格的ROSE细胞涂片标本进行了相同的检测。在这10例配对标本中，石蜡组织中检测到的所有驱动突变均在其配对的ROSE细胞涂片标本中检测到。44例石蜡组织不合格但其配对的ROSE细胞涂片合格标本中，8例提取的DNA量<10 ng，25例DNA量为10-50 ng，11例提取的DNA量>50 ng，DNA量>50 ng的11例标本进行了NGS检测，11例标本均检测到体细胞突变，其中9例检测到驱动基因突变（表4）。DNA量为10-50 ng的25例标本进行了肺癌9个基因突变实时定量PCR联合检测，其中15例检测到驱动基因突变（表5）。

**表4:** FFPE切片组织不合格但其配对ROSE细胞涂片合格标本NGS突变详细情况

Case No.	Tumor cell number and proportion		DNA yield of ROSE smears (ng)	Mutation detected on ROSE smears
	FFPE tissue section	ROSE smears		
1	150; 5%	3000; 50%	76.3	EGFR Ex21 L858R: 31.8% TP53 Ex8 R273C: 19.4%
2	150; 5%	3000; 30%	55.1	KRAS Ex2 G13C: 41.0% MET amplification: 4.6% TP53 Ex7 R248W: 7.3% NF1 IVS13: 36.3% CDKN2A Ex2 H83Y: 23.5%
3	200; 5%	3000; 50%	67.6	TP53 Ex4 A78Sfs*70: 30.1% JAK2 Ex3 Y68N: 23.4%
4	200; 5%	4000; 30%	52.0	EGFR Ex19 deletion: 13.2% MDM2 amplification: 8.6% CDK4 amplification: 4.0%
5	200; 5%	4000; 40%	50.5	KRAS Ex2 G12V: 37.0% TP53 Ex5 A159V: 38.2%
6	50; 1%	5000; 60%	86.8	EGFR Ex21 L858R: 45.8%
7	50; 1%	3000; 30%	54.1	KRAS Ex2 G13C: 33.2% TP53 Exon5 G154V: 25.2% BRIP1 Ex11 G492*: 8.1% B2M Ex1 deletion: 4.5%
8	100; 5%	4000; 40%	56.7	EML4-ALK fusion: 18.9%
9	100; 2%	6000; 60%	74.4	KRAS Ex2 G12C: 29.4% TP53 Ex5 R158L: 22.9%
10	200; 5%	5000; 40%	51.4	EGFR Ex19 deletion: 24.2% EGFR Ex20 T790M: 7.5% MDM2 amplification: 5.0%
11	150; 5%	5000; 50%	55.2	EGFR Ex21 L858R: 32.2% EGFR Ex21 L833V: 31.9% TP53 Ex6 H214R: 16.9%

NGS: next-generation sequencing; EGFR: epidermal growth factor receptor; Ex: exon; TP53: tumor protein 53; KRAS: kirsten rat sarcoma viral oncogene; MET: mesenchymal-epithelial transition factor; NF1: neurofibromatosis type 1; JAK2: janus kinase 2; MDM2: murine double minute 2; CDK4: cyclin-dependent kinase 4; B2M: beta-2-microglobulin; EML4: echinoderm microtubule associated protein like 4; ALK: anaplastic lymphoma kinase; *: stop codon.

**表5:** FFPE切片组织不合格但其配对ROSE细胞涂片合格标本PCR检测突变详细情况

Case No.	Tumor cell number and proportion		DNA yield of ROSE smears (ng)	Mutation detected on ROSE smears
	FFPE tissue section	ROSE smears		
1	50; 1%	1000; 20%	13.4	Negative
2	50; 1%	1200; 30%	12.8	EGFR Ex21 L858R
3	100; 5%	1000; 20%	11.4	KRAS Ex2 G12A
4	100; 3%	1500; 30%	15.7	ALK Ex19, 20 fusion
5	100; 5%	1000; 20%	10.3	EGFR Ex21 L858R
6	150; 5%	1200; 30%	14.2	Negative
7	150; 3%	1200; 30%	12.5	Negative
8	150; 5%	1500; 30%	14.3	Negative
9	300; 5%	1500; 30%	17.5	EGFR Ex20 S768I;EGFR Ex21 L858R
10	150; 5%	1200; 50%	14.4	EGFR Ex21 L858R
11	50; 1%	1500; 50%	16.5	ROS1 Ex32, 34 fusion
12	150; 5%	1500; 30%	15.2	KRAS Ex2 G12A
13	100; 5%	1800; 50%	18.0	Negative
14	200; 5%	1800; 30%	11.2	EGFR Ex19 deletion
15	30; 1%	2000; 50%	17.8	Negative
16	50; 1%	2000; 50%	15.2	Negative
17	100; 3%	2000; 50%	16.9	EGFR Ex19 deletion
18	100; 3%	2000; 30%	21.4	EGFR Ex21 L858R
19	100; 10%	2000; 30%	26.2	KRAS Ex2 G12A
20	100; 3%	2000; 20%	19.3	KRAS Ex2 G12D
21	150; 2%	2000; 30%	18.6	PIK3CA Ex9 E545L;PIK3CA Ex20 H1047R; EGFR Ex19 deletion
22	150; 5%	2500; 50%	24.3	Negative
23	150; 5%	2500; 30%	48.3	Negative
24	150; 5%	2500; 50%	35.7	Negative
25	300; 5%	2500; 50%	21.6	KRAS Ex2 G12A

PCR: polymerase chain reaction.

## 3 讨论

相关驱动基因突变的分子检测已成为多种癌症的标准治疗方案。随着靶向治疗药物种类的增加，临床对小样本（包括细胞学标本和小活检）辅助分子检测的需求将持续增长。对于肺癌尤其如此，因为许多肺癌患者就诊时已经是疾病晚期，无法进行手术治疗。我们经常面临在相对较少的样本上进行分子检测的挑战。目前，所有肿瘤标本的病理诊断方法，包括形态分型、免疫组织化学和分子分型几乎全是依赖于FFPE组织切片。其他形式的标本，例如直接涂片、液基细胞学涂片及ROSE细胞学涂片被利用的程度不高。如何通过微创手术获得足量标本以进行组织学诊断和分子分型，从而精准指导后续治疗，是临床医师和病理科工作人员面临的关键问题。

组织印片或针吸细胞学涂片结合迪夫快速染色法的ROSE技术已逐步发展成为介入学科的重要辅助手段，特别是在呼吸系统疾病的诊断中。ROSE可以对标本进行现场解读，迅速给出初步诊断，并对下一步操作给予指导。ROSE优化了常规支气管镜、EBUS-TBNA和经皮肺穿刺等活检手段的诊断效能和准确性。在本研究中，我们发现大部分NSCLC患者TBLB及EBUS-TBNA的ROSE细胞学涂片有丰富的肿瘤细胞，利用合格的ROSE涂片进行分子检测的准确性等同于或优于石蜡组织标本。它可以作为石蜡组织标本的重要的补充标本，也可以与患者的石蜡组织标本或其他细胞学标本结合使用。

Harada等^[7]^研究表明，单独采用细胞蜡块时，针吸标本的分子检测合格率仅为36%，增加迪夫快速染色的细胞学涂片标本后，分子检测合格率提高到68%。Fielding等^[8]^研究表明EBUS-TBNA标本的迪夫快速染色的细胞学涂片提取的DNA量及分子检测合格率比对应的组织蜡块高，细胞涂片与石蜡切片标本检出的突变数量相当。33例配对标本中，共有10例检出驱动基因突变，其中8例在细胞涂片和石蜡切片标本中均有检出，1例仅在细胞涂片标本中检出，1例仅在石蜡切片标本中检出；有24例共检出41个非驱动基因突变，其中20例的29个非驱动基因突变在细胞涂片和石蜡切片均检测出，3例的8个非驱动基因突变仅在细胞涂片中检出，4例的4个非驱动基因突变仅在石蜡切片中检出。有研究^[8]^认为肿瘤细胞数量>1000且肿瘤细胞比例≥25%的细胞涂片标本预示可以获取高量的DNA并成功进行NGS，本研究也证实了这个观点。

本研究发现，与石蜡切片相比，采用ROSE细胞涂片进行分子检测有三方面的优势：（1）从细胞涂片提取DNA在操作步骤上可省去石蜡包埋、切片、刮片、脱蜡等过程，可减少分子检测流程的时间。（2）ROSE细胞涂片具有“所见即所得”的优点，载玻片上观察到的肿瘤细胞都可用来提取DNA，评估的肿瘤细胞量能更好地预示后续分子检测的成功。而石蜡组织只能通过观察表面的一张切片进行肿瘤细胞量的评估，这并不能真实反映深层组织连续多次切片后的肿瘤细胞量。（3）组织在福尔马林固定过程中，核酸与福尔马林形成加和物，影响DNA质量，导致获得的DNA量明显减少，并且为片段化的DNA。而细胞涂片标本未经福尔马林固定，能获得更高质量的DNA^[16,17]^。另外，石蜡组织的4-5 μm切片只能代表细胞的一个切面，不能代表完整的细胞，而细胞涂片为完整的细胞。所以从细胞涂片提取的DNA质量优于石蜡切片提取的DNA，分子检测要求的最低DNA量减少5-8倍时仍可获得满意结果^[18]^。目前各种分子检测平台包括实时定量PCR法及NGS法，标本合格的要求不仅包括最低肿瘤细胞量还包括最低肿瘤细胞比例。肿瘤细胞量或肿瘤细胞比例过低都可导致假阴性结果^[11,19⇓⇓-22]^。因此，在分子检测标本的选择过程中，从肿瘤细胞比例方面考虑，一些病例的ROSE细胞涂片标本会优于其对应的石蜡切片标本。应用ROSE细胞涂片作为替代的样本进行分子检测后，相应的石蜡切片标本可用做更合适的检测，例如免疫组化检测ALK、ROS1和细胞程序性死亡配体1（programmed cell death ligand 1, PD-L1）的表达，荧光原位杂交（fluorescence in situ hybridization, FISH）检测基因融合、重排或基因扩增。

Tong等^[23]^研究了肺芯针穿刺标本的ROSE细胞涂片和配对石蜡切片肿瘤细胞量，发现ROSE的组织印片技术可影响后续石蜡组织的肿瘤细胞量，导致部分病例的ROSE细胞涂片上黏附有较多的肿瘤细胞，而石蜡组织切片仅有少量或无肿瘤细胞残留。进一步的体外实验^[24]^证实，印片操作过程中太过用力或组织拖行过长，可导致石蜡组织提取的DNA量减少25%以上。因此ROSE印片操作过程中要避免用力过大，以免耗竭组织中的肿瘤细胞。本研究发现，采用组织印片的TBLB标本有相当一部分病例存在石蜡切片肿瘤细胞量少而ROSE细胞涂片肿瘤细胞量多的情况。而采用涂片的EBUS-TBNA标本这种情况较少。因此选择分子检测标本时要注意综合考虑石蜡组织和细胞涂片这两种标本，单选用石蜡组织而不采用ROSE细胞涂片可能导致部分患者无足够的标本进行分子检测，特别是采用组织印片技术的TBLB活检标本。

本研究的不足之处在于，我院呼吸介入科只在部分TBLB及EBUS-TBNA小活检术中行ROSE检测，本研究为回顾性研究，收集的为非连续性病例，有可能存在抽样偏差。未来有必要开展前瞻性的连续病例研究，全面地评估ROSE操作对小活检标本石蜡切片的影响及ROSE细胞涂片对分子检测的补充作用。另外，本研究仅对10例配对的石蜡切片和ROSE细胞涂片标本进行分子检测，初步说明ROSE细胞涂片与石蜡切片分子检测的结果相同。有必要增加配对样本的分子检测，进一步明确这两种样本分子检测的敏感性、特异性。

本研究表明，部分行术中ROSE的病例存在石蜡切片标本肿瘤细胞量较少，而其细胞涂片标本肿瘤细胞较丰富。合格的ROSE细胞涂片进行分子检测的准确性等同于或优于石蜡组织标本。采用ROSE细胞涂片补充石蜡切片标本进行分子检测增加了小活检标本分子检测的标本合格率，增加了靶向药物的分子标志物的发现，为更多的患者选择靶向治疗提供了机会。建议选择分子检测标本时综合考虑石蜡组织和细胞涂片这两种标本。

**Competing interests**

The authors declare that they have no competing interests.

**Author contributions**

Jiang JH conceived and designed the study. Tang SQ and Lin ZY performed the experiments. Tang SQ analyzed the data. Tang CL provided study material or patients. Jiang JH and Tang CL provided critical inputs on design, analysis, and interpretation of the study. All the authors had access to the data. All authors read and approved the final manuscript as submitted.
